# PAM50 Intrinsic Subtypes and Immunity Status in Prognosis of Triple-Negative Breast Cancer: A Retrospective Cohort Study

**DOI:** 10.3390/cancers17244010

**Published:** 2025-12-16

**Authors:** Yuan Wang, Yu Song, Songjie Shen, Huanwen Wu, Xinyu Ren, Zhiyong Liang

**Affiliations:** 1 Department of Pathology, State Key Laboratory of Complex Severe and Rare Disease, Molecular Pathology Research Center, Peking Union Medical College Hospital, Chinese Academy of Medical Sciences and Peking Union Medical College, Beijing 100730, China; 2 Department of Breast Surgery, Peking Union Medical College Hospital, Chinese Academy of Medical Science and Peking Union Medical College, Beijing 100730, China

**Keywords:** triple negative breast cancer, PAM50 intrinsic subtype, immunity score, adjuvant chemotherapy, PD-L1 expression level

## Abstract

**Background:** Although triple-negative breast cancer (TNBC) patients commonly receive adjuvant chemotherapy after surgery, their prognoses vary. This study aimed to investigate how the intrinsic subtypes of TNBCs and immune status of patients affect their prognosis. **Methods:** A total of 111 TNBC patients were retrospectively analyzed at Peking Union Medical College Hospital from 2002 to 2014. All underwent surgery and received adjuvant chemotherapy per NCCN guidelines. Intrinsic subtypes (luminal A, luminal B, HER2-enriched, and basal-like) were identified using PAM50 profiling. Recurrence-of-risk (ROR) scores were classified into high, intermediate, and low. Immune status was assessed via a 17-gene panel and categorized as immune-strong or immune-weak. Statistical analyses included chi-square tests, Kaplan–Meier curves, log-rank tests, and Cox regression. **Results:** All four PAM50 subtypes were present, with basal-like being the most common (77%). Luminal A patients with low-to-intermediate ROR scores showed worse outcomes than other subtypes (DFS, *p* = 0.123; OS, *p* = 0.170). Unexpectedly, high-ROR patients had the longest DFS (*p* = 0.042). Immune-strong status correlated with improved DFS and OS in stage IIB–III patients (DFS, *p* = 0.029; OS, *p* = 0.003), and was associated with higher TILs (*p* = 0.015) and PD-L1 expression on tumor cells (*p* = 0.022). **Conclusions:** Multigene-based assessment of molecular subtype and immune status provides important prognostic insight into TNBC and may guide adjuvant treatment decisions, particularly in non-basal-like subtypes.

## Simple Summary

Triple-negative breast cancer is a challenging type of breast cancer because it lacks common treatment targets and shows very different outcomes among patients. Doctors often rely on standard chemotherapy, but not all patients benefit equally. This study explored whether two biological features—the internal molecular type of tumor and the strength of the patient’s immune response—could help predict which patients are more likely to do well after treatment. By analyzing tumor tissues from more than one hundred patients, the study found that some molecular types respond better to chemotherapy, and patients with stronger immune activity tend to have better survival. When these two features are combined, they provide a clearer picture of prognosis. These findings may help doctors in the future personalize treatment decisions and identify patients who might benefit from additional or alternative therapies.

## 1. Introduction

Triple-negative breast cancer (TNBC), which accounts for 10–15% of all breast cancers, lacks amplification of estrogen receptors (ERs), progesterone receptors (PRs), and human epidermal growth factor receptor-2 (HER2) [1]. Traditionally, chemotherapy has been the only method used to treat TNBC patients. Patients diagnosed with TNBC have a higher risk of disease relapse within 5 years than those diagnosed with other breast cancer subtypes due to its high heterogeneity [2]. In addition to standard anthracycline- and taxane-based regimens, alternative chemotherapies such as paclitaxel plus carboplatin have been investigated for their efficacy in TNBC patients [3].

Molecular portraits of breast cancer are envisioned to provide a rationale for breast cancer prognosis and the prediction of therapy to discover actionable targets and drugs. Four major classifications of TNBC in whole-genome gene expression profiling (GEP) studies were identified: intrinsic PAM50 and the molecular subsets described by Burstein et al. [1], Lehmann et al. [2], and Jiang Y.Z. [3]. None of them have been widely used in clinical practice. The PAM50 assay (Prosigna Breast Cancer Gene Sig-nature Assay, NanoString Technologies) contains the lowest number of genes and is the most easily used assay [4]. It was approved by the U.S. Food and Drug Administration (FDA) and included in the American Joint Committee on Cancer (AJCC) guidelines to guide the treatment and risk assessment of ER-positive breast cancer. However, this method has not been widely implemented in China due to its high cost and being time-consuming. In routine pathological studies, breast cancer subtypes are classified using four immunohistochemistry (IHC) markers (ER, PR, HER2, and Ki-67) to replace the gene subtype while designing treatments, predicting patient prognosis, and even enrolling in clinical trials. However, the IHC surrogate allowed us to use the words “TNBC” and “basal-like” interchangeably. To date, few data are available regarding the prognostic impact of intrinsic molecular subtypes of TNBC defined using the PAM50 after chemotherapy. PAM50 assay was used to evaluate TNBC to find useful prognosis tips and treatment strategies.

Furthermore, TNBC exhibits elevated infiltration of immune cells, antigen presenting cells, and activation of immune response pathways [5,6]. In addition, immune checkpoint inhibitors have yielded promising results in patients with advanced and early-stage TNBC. The manipulation of the immune system is an attractive strategy for treating TNBC. It has previously been demonstrated that 17 immunity genes have significant prognostic stratification in ER-negative breast cancer [7].

Therefore, this study aimed to assess the discordance between the IHC- and PAM50-based intrinsic subtypes and to observe how intrinsic subtypes and tumor immune status affect the prognosis of TNBCs after adjuvant chemotherapy.

## 2. Materials and Methods

### 2.1. Patients and Clinical Information

A total of 111 patients diagnosed with TNBC at Peking Union Medical College Hospital (PUMCH) between 2002 and 2014 were eligible for this study. All patients underwent surgery according to NCCN guidelines, including breast-conserving surgery or mastectomy combined with sentinel lymph node biopsy (SLNB) or axillary lymph node dissection (ALND), as clinically indicated. Postoperative adjuvant chemotherapy consisted of anthracycline- and taxane-based regimens, administered sequentially or in combination, for 4 to 8 cycles. Patients undergoing breast-conserving surgery, or patients with more than 3–4 axillary lymph node metastases, or with T4 tumor received local radiotherapy. All adjuvant treatment regimens followed NCCN guidelines at the time of diagnosis.

Clinical information was collected for analysis, including age, staging, tumor size, date of diagnosis, date of local recurrence, distant metastasis or death, and date of the last follow-up. Those patients treated with neoadjuvant chemotherapy before operation and those who provided insufficient tissue samples were excluded. The follow-up period was from the surgery date to 31 March 2019. The median follow-up period was 92 months. Overall survival (OS) was defined as the time from diagnosis to the time of death from the disease. Disease-free survival (DFS) was defined as the time from diagnosis to the first relapse of the disease (local, regional, distant metastasis, or contralateral breast cancer). Disease recurrence and metastases were confirmed using diagnostic imaging and pathology.

The study was approved by the Institutional Review Board of PUMCH. Informed consent was obtained from all the patients.

### 2.2. Histopathological Tumor-Infiltrating Lymphocyte Assessment

Tumor-infiltrating lymphocytes (TILs) were evaluated on Hematoxylin and Eosin (HE) stained slides by two experienced pathologists according to previously described methods [8]. The scores used for each patient were 0 for virtual absence, 1 for low (<30%), 2 for moderate (30–60%), and 3 for high (>60%) TILs. Cases that could not be appropriately evaluated for technical reasons (such as bad staining or low tumor area) were designated as “not evaluable”. In the case of inter-observer discordance in TIL abundance, a particular slide was reviewed jointly, and an agreeable score was assigned.

### 2.3. Immunohistochemistry Staining and Result Interpretation

Sections (4 mm thick) were mounted on adhesion slides from tissue microarray blocks. IHC staining was performed using a Ventana Benchmark XT autostainer (Ventana Medical Systems Inc., Tucson, AZ, USA) according to the manufacturer’s protocol. Antibodies against ER, PR, HER2, EGFR, CK14, CK5/6, P53, and Ki-67 were stained on each tissue at the diagnosis time, and PD1, PD-L1 was stained on the tissue at the start of the experiment. The respective interpretation criteria were the same as those described previously [9]. TNBC was defined as negative for ER, PR was defined as <1% nuclear staining using immunohistochemistry (IHC), and HER2 was defined as 0 or 1 using IHC or non-amplified using fluorescent in situ hybridization (FISH).

### 2.4. Targeted RNA Expression Detection Using RNA-Seq

For breast tumor FFPE tissues, RNA extraction was routinely performed using an RNA Storm RNA extraction kit (CD201, CELLDATA, Fremont, CA, USA), according to the manufacturer’s protocol. The Illumina TruSeq Targeted RNA Expression Kit was used to build libraries of the targeted genes, as previously described. Briefly, cDNA was synthesized from purified RNA using ProtoScrip II Reverse Transcriptase. The cDNA was hybridized with custom oligo pools in a thermal cycler programmed to decrease temperature gradually (from 70 °C to 30 °C). The RNA/oligo hybrid products were washed, extended, and ligated. The ligated DNA was amplified using DNA polymerase in a thermal cycler (from 95 °C to 10 °C). The PCR products were purified using AMPure XP beads and analyzed using Agilent Bioana-lyzer2100 and DNA1000 chips for quality control. According to the manufacturer’s plan, equal amounts of mixed samples from each sample library are loaded onto iSeq100 for sequencing. Illumina Casava1.7 software is used for base calling, and Illumina bcl2fastq2 software is used for demultiplexing sequencing data to generate a FASTQ file for each sample. Only samples with total reads > 10000 and missing genes < 10% were further validated, in order to ensure the integrity of sequencing data from libraries derived from FFPE RNA tissue. Sequencing reads from Read 1 of each FASTQ file were aligned to the target genomic regions, and raw counts were generated using the R package ShortRead (version 1.48.0). The raw counts of all samples were normalized based on the size of transcripts and libraries, and then the counts per million (CPM) of each sample were calculated using Bioconductor’s R-packaging edge R as the gene expression matrix. The k-nearest neighbor (KNN) method was used to impute missing values. Gene expression data were then log2-transformed, median-centered, and standardized.

The positivity of ER, PR, or HER2 at the RNA level was determined using a database of 1951 breast cancer patients with whole transcriptomic profiles matched with the IHC or FISH results of ER, PR, and HER2 [7]. Expression of the three genes was normalized and scaled to a 0–100 score, with 0 indicating no expression and 100 indicating the highest expression. ER, PR, and HER2 cutoff values were 38, 51, and 63, respectively.

### 2.5. Intrinsic Subtype Identification and Calculation of Relapse (Risk of Recurrence) and Immunity Scores

Intrinsic subtype analysis based on expression profiles was performed using the PAM50 predictor and applied to the nearest PAM50 centroid algorithm Bioclassifier to identify the PAM50 intrinsic subtypes, as described by Parker et al. [4]. Samples were classified into the following intrinsic subtypes: luminal A, luminal B, HER2-enriched, and basal-like. Normal-like data were excluded from further analyses because of potential contamination from the normal breast tissue. There was no normal-like subtype case in our cohort. The risk of recurrence (ROR) score (0–100) was a value to predict the risk index of distant metastasis based on the distribution of PAM50 molecular subtypes and proliferation index and calculated as described previously [10]. Patients were assigned to low-, medium-, and high-risk groups according to ROR scores (high > 60, middle 40–60, and low < 40; the cutoff values come from the original PAM50 research) [10]. An immunity score based on the expression profile of 17 immunity genes was calculated and scaled as 0–100 according to the method published by Yang et al. [7]. Immunity scores between 35 and 60 were evaluated for sensitivity Scheme S1) that best separates immune status according to the method published by Yang et al. [7], and the patients were divided into two groups, “i-weak (<40)” and “i-strong (≥55)”, based on their immunity scores.

### 2.6. Statistical Analysis

Statistical analysis was performed using SPSS 17.0 (SPSS, Chicago, IL, USA). Qualitative variables were compared between immune status groups using the chi-square test. The clinicopathological characteristics analyzed included age, tumor size, histological grade, P53 status, Ki-67 index, PAM50-based proliferation score, IHC-based and PAM50-based basal-like subtype classification, PD-L1 expression on tumor cells and TILs, PD-1 expression on TILs, TIL density, and lymph node metastasis. The concordance between IHC and PAM50 basal-like subtypes was measured by kappa test. DFS was measured from the date of curative surgery to the date of the first recurrence. OS was measured from the date of curative surgery to the date of death or last follow-up. A false discovery rate was applied for multiple testing corrections. The Kaplan–Meier method was used to estimate DFS and OS, and survival curves were compared using the log-rank test. A Cox proportional hazard regression model was used to assess the impact of the prognostic variables on OS. All tests were two-tailed, and statistical significance was set at *p* < 0.05. A post hoc power analysis of all results was performed to exclude the influence of sample size differences between subgroups (Scheme S2).

## 3. Results

### 3.1. Patient Characteristics

The patient characteristics are shown in Table 1. A total of 111 patients with TNBC were analyzed. The median age of all patients was 49 years (range, 25–90 years), and approximately 51.4% were premenopausal. Furthermore, 97.3% (108/111) of the patients had invasive carcinoma of no special morphology, with the majority (74.8%) having a high histological grade. All the patients underwent curative surgery. Thirty-seven (33.3%) patients presented with distant metastases, and seven (6.3%) had ipsilateral local recurrence. Twenty-eight (25.2%) patients died of the disease during the follow-up. The follow-up duration ranged from 7 to 182 months, with a median of 92 months.

### 3.2. Prognosis of TNBC with Different Intrinsic Subtypes, ROR, and Immunity Scores After Adjuvant Chemotherapies

**Figure 1:**
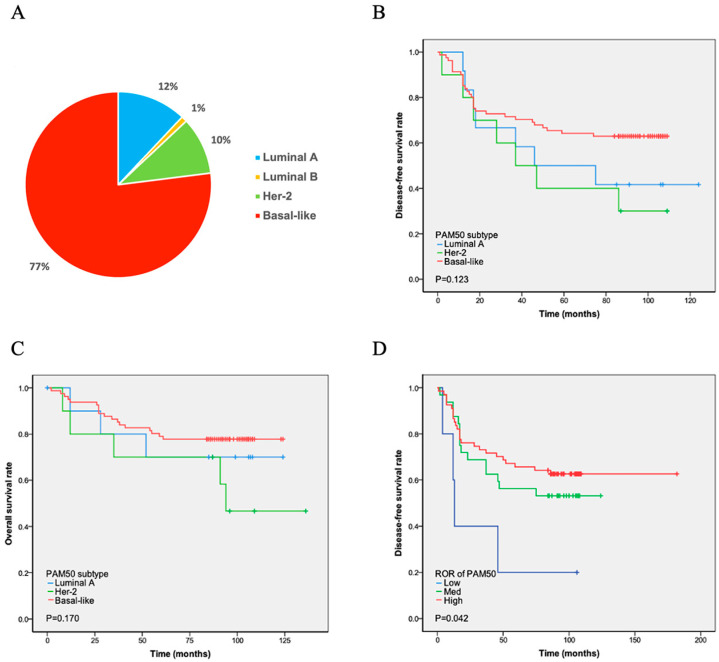
PAM50 subtype distribution and survival analyses. (**A**) Proportion of patients: Basal-like subtype showed the highest prevalence (77%), while luminal B subtype had the lowest (1%). (**B**,**C**) Disease-free (DFS) and overall survival (OS): No statistically significant differences (*p* > 0.05) were observed in DFS (**B**) or OS (**C**) between luminal A, HER2-positive, and basal-like subtypes. (**D**) DFS after chemotherapy by ROR: Patients identified as high-risk by PAM50 subtype prediction had the longest DFS following chemotherapy, whereas low-risk patients had the shortest DFS. Note: The unexpected better DFS in the high-ROR patients might be because they were more chemotherapy sensitive.

PAM50 analysis revealed four intrinsic subtypes of TNBC. Among them, the basal-like (*n* = 86, 77%) subtype was most frequently observed, followed by luminal A (*n* = 13, 12%), HER2-enriched (*n* = 11, 10%), and luminal B (*n* = 1, 1%) subtypes (Figure 1A). Among the 13 patients with luminal A, 10 were ER- and/or PR-positive at the RNA level. Three of the eleven HER2-enriched patients were HER2-positive at the RNA level (Table 2). The intrinsic subtypes showed a significantly different prognosis for relapse-free survival and OS in patients with TNBC. Luminal B was not subjected to Cox regression analysis due to the limited number of cases. The K-M analysis showed that the remaining three subtypes had similar curves in DFS and OS. The basal-like subtype, which has the worst prognosis in ER-positive breast cancer, demonstrated longer DFS (*p* = 0.123) and OS (*p* = 0.170) times than the luminal A and HHER2 subtypes. In contrast, the HEHER2 subtype had the shortest DFS and OS times (Figure 1B and Figure 1C, respectively).

The PAM50 system divides ROR into three levels: high, middle, and low. The ROR of luminal A cases was low in five patients and medium in eight patients (Table 2). Six HER2-enriched cases were at medium risk, and five were at high risk. Furthermore, 74.4% (64/86) of the patients with the basal-like subtype were at high risk, as evaluated using PAM50 ROR, 24.4% (21/86) were at medium risk, and 1.2% (1/86) were at low risk. The ROR detected using PAM50 showed that high-risk patients had the longest DFS, whereas low-risk patients had the shortest DFS (*p* = 0.042; Figure 1D).

**Figure 2:**
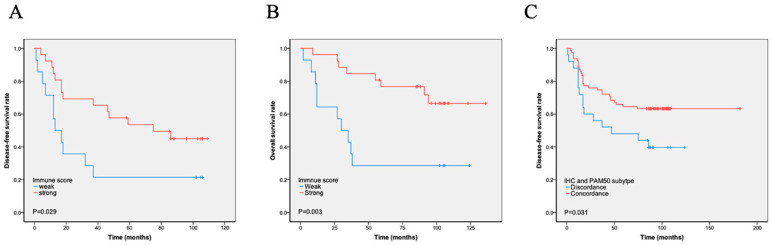
Kaplan–Meier analysis of immune scores and PAM50–IHC concordance on survival. (**A**–**B**). Immune score and survival: Patients classified as immune-strong by immune score analysis had significantly longer disease-free survival (DFS) (**A**) and overall survival (OS) (**B**) compared to those classified as immune-weak. (**C**) PAM50–IHC concordance and DFS: Patients with concordant PAM50 intrinsic subtype and immunohistochemistry results exhibited significantly longer DFS compared to those with discordant results.

The immunity score was calculated using the expression of 17 immune-related genes, and patients were divided into i-strong and i-weak groups based on the immunity scores. A total of 76 (68.5%) cases were i-strong, whereas 35 (31.5%) were i-weak. We found that i-strong was associated with a better prognosis, especially in stage IIB and III cases (DFS, *p* = 0.029, Figure 2A; OS, *p* = 0.003, Figure 2B). Immune scores varied significantly across PAM50 molecular subtypes, with luminal A tumors showing a higher immune score compared with basal-like tumors, as detailed in Table S1. Within the basal-like subtype, however, survival did not differ significantly between immune-strong and immune-weak groups, as shown in Scheme S3. The luminal A subtype had the highest proportion of i-strong cases (92.3%), followed by the HER2-enriched (54.5%) and basal-like (48.8%) subtypes. The only luminal B case had a high immune score. High immune gene expression was also significantly negatively correlated with proliferation score (*p* = 0.000) and Ki-67 index (*p* = 0.016) but positively correlated with the number of TILs (*p* = 0.015) and tumor cells’ PD-L1 expression (*p* = 0.022). High immune gene expression was also significantly associated with the PAM50-based molecular basal-like subtype compared with the IHC-based basal-like subtype. There was no significant association between i-strong and other characteristics, such as age, tumor size, stage, P53, and lymph node positivity (Table 3). However, multivariate analyses showed that immune score was not an independent prognostic factor (Table 4 and Table 5, Tables S4 and S5).

### 3.3. Discordance in the Identification of Intrinsic Subtypes Between IHC and PAM50

Based on the expression of basal-like markers, such as EGFR, CK5/6, and CK14, TNBC could be divided into basal-like and non-basal-like subtypes using IHC [11]. IHC detection showed 96 (86.5%) cases of the basal-like subtype and 15 (13.5%) cases of the non-basal-like subtype. PAM50 testing found 86 cases of the basal-like subtype and 25 cases of the non-basal-like subtype. Among them, 78 cases were consistent with histological and PAM50 testing, while 26 cases were inconsistent. Among them, 18 cases were histologically classified as basal-like, but not detected by molecular testing. Further analysis showed that 11 IHC basal-like cases were PAM50 HER2 subtypes and 7 cases were luminal A subtypes. Among them, all cases with Ki-67 < 30% were luminal A subtypes (Tables S2 and S3). Eight (8/86, 9.3%) cases were detected as basal samples by molecular testing but did not express immunohistochemical markers. Totally, IHC subtyping of 85 cases was consistent with PAM50 subtyping, accounting for 76.6% of all cases. The concordance between IHC and PAM50 basal-like subtypes was low (kappa = 0.218). To evaluate the impact of discordance on survival, we analyzed DFS and OS according to discordance. Patients with the concordant subtype had significantly longer DFS (*p* = 0.031) and OS (*p* = 0.290) than those with the discordant subtype (Figure 2C).

### 3.4. Combining PAM50 Intrinsic Basal-like Subtypes and Immune Status Could Predict Prognosis

**Figure 3:**
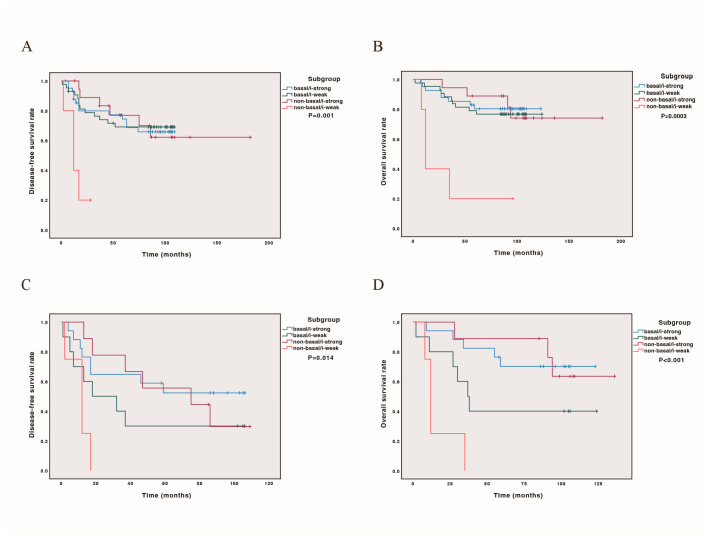
Kaplan–Meier analysis combining PAM50 basal-like and immune scores on survival. Patients classified as base-like/i-strong, basal-like/i-weak, non-basal-like/i-strong, non-basal-like/i-weak. (**A**) DFS curve in 111 cases; (**B**) OS curve in 111 cases; (**C**) DFS curve in 43 cases with stage later than IIB; (**D**) OS curve in 43 cases with stage later than IIB.

We further conducted DFS and OS curves for all cases and cases later than stage IIB, incorporating PAM50 subtype classification (basal-like or non-basal) and immune score status. Our findings revealed that both in all cases and in cases later than stage IIB, the non-basal/i-weak subtype had the poorest prognosis (*p* = 0.001, 0.014 for DFS, Figure 3A,C; *p* = 0.0003, *p* < 0.001 for OS, Figure 3B,D). The prognostic stratification was more pronounced in cases beyond stage IIB, with outcomes ranking from best to worst as follows: non-basal/i-strong, basal/i-strong, basal/i-weak, and non-basal/i-weak (Figure 3). Detailed survival estimates and post hoc comparisons are provided in Scheme S4.

## 4. Discussion

All four PAM50 subtypes (luminal A, luminal B, HER2-enriched, and basal-like) were detected in TNBC patients, among which the basal-like subtype was the majority (77%). This is similar to the results of Lehmann’s (80.6%) study [12]. Burstein’s basal-like immuno-suppressed (BLIS) and basal-like immune-activated (BLIA) subtypes were entirely basal-like, accounting for 86% and 74% of all PAM50 basal-like tumors in the discovery and validation sets, respectively [1]. Lehmann et al. demonstrated that TNBC subtypes differed significantly in their response to similar neoadjuvant chemotherapy [13]. Lehmann’s research showed that the basal-like subtype was most sensitive to chemotherapy. In a retrospective analysis of 367 samples, a greater benefit of bevacizumab was observed in treating basal-like disease (that is, pCR increased from 45% to 64%, *p* ¼ 0.0009) but not in treating non-basal-like disease (that is, pCR decreased from 60% to 43%; *p* ¼ 0.024) [14]. Similarly, Gluz O et al. found that in early TNBC, basal-like subtype, higher Ki-67 (IHC), and lower HER2 score were associated with chemosensitivity [15]. We found that different subtypes classified using the PAM50 also had very different prognoses after chemotherapy. Among the four subtypes based on PAM50, the basal-like subtype had the best prognosis, the luminal A subtype had the second-highest prognosis, and the HER2 subtype had the worst prognosis. This indicated that the basal-like subtype benefited the most from chemotherapy, which was similar to previous studies [14,15]. Notably, among the 13 patients with the luminal A subtype, 10 were ER- and/or PR-positive at the RNA level. In ER-positive breast cancer, luminal A showed the lowest pCR rate of neoadjuvant chemotherapy and a higher benefit from adjuvant tamoxifen [16,17,18]. Therefore, endocrine therapy might benefit the PAM50’s luminal A subtype of TNBC. Three of the eleven HER2-enriched patients were HHER2-positive at the RNA level. For the same reason, the HER2-enriched subtype may need additional corresponding target therapy.

The ROR of the PAM50 level, which is useful for predicting the prognosis of ER-positive breast cancer, showed a contrary prediction. High-risk patients had the longest DFS and OS, whereas low-risk patients had the shortest DFS (*p* = 0.042) and OS (*p* = 0.517). The ROR score based on PAM50 is not solely dependent on molecular typing, but rather a comprehensive score. It is calculated from three parts of information, including PAM50 typing, proliferation-related level expression, tumor size, and lymph node status. The clinical significance was combining endocrine therapy with chemotherapy for HR+/HER2 patients at high risk of ROR. All our cases of TNBC patients underwent chemotherapy after surgery. This proved that the survival rate in the high-risk group of ROR is highest after chemotherapy. A total of 89.6% (60/67) of high-risk ROR patients belong to the basal-like subtype, indicating that the basal-like subtype is more sensitive to chemotherapy. Though our investigation found that PAM50 ROR could predict TNBC prognosis, its predicting value needs to verify in larger samples and a more precise recurrence score needs to be found for TNBC.

In routine pathological studies, breast cancer subtypes are classified using IHC. IHC basal-like TNBC was identified by the expression of basal-like markers, such as CK5/6 and EGFR [19]. The consistency between IHC and PAM50 results was low (kappa = 0.218). Patients with the concordant subtype had significantly longer DFS (*p* = 0.031) and OS (*p* = 0.290) than those with the discordant subtype. This result indicated that only the gene expression-based basal-like subtype benefited from existing chemotherapy programs. Patients whose tumors are classified as non-basal by IHC but basal-like by PAM50 may still derive substantial benefit from chemotherapy, suggesting that reliance on IHC alone could potentially underestimate chemosensitivity in these cases. Conversely, misclassification could lead to under- or overtreatment if treatment decisions were based solely on IHC. Our study also shows that 90.7% (78/86) of PAM50 basal-like subtypes can be recognized by IHC. In conditions that molecular testing could not be reached, IHC remains an effective alternative detection tool.

Apart from the molecular subtype, the immune state is another factor that remarkably affects the prognosis and effect of chemotherapy in TNBC patients. Regarding Lehmann’s classification, the immunomodulatory group showed a relatively better outcome [2,12,13]. Burstein et al. reported that only the basal-like immune-activated (BLIA) group showed better outcomes than the others [1]. The PAM50 molecular subtyping of TNBC could indicate different prognoses; however, it did not include immune-related genes. Immune gene signatures were used to predict the relapse of TNBC in studies [20]. Therefore, we further detected a cluster of 17 immune genes that showed prognostic value in TNBC and predicted their value in response to neoadjuvant chemotherapy. Our results showed that 76 of 111 (68.5%) cases were i-strong. As expected, high immune gene expression was associated with a better prognosis. In cases with a stage later than IIB, the difference was significant (DFS, *p* = 0.029; OS, *p* = 0.003). While immune score was not an independent prognostic factor in multivariate analyses, possible explanations include confounding by other strong prognostic signatures such as PD1 expression of TILs and TIL scores higher than 2. Lymph node metastasis may reduce the apparent independent contribution of the immune score. We note that larger cohorts and prospective studies are needed to disentangle these effects and formally evaluate the independent prognostic value of immune metrics, ideally using combined models that integrate both intrinsic subtype and immune activity.

Analysis of immune state and other clinicopathological characteristics showed that high immune gene expression was significantly positively correlated with the number of TILs (*p* = 0.015) and molecular basal-like subtypes (not IHC basal-like subtype) and negatively correlated with proliferation score (*p* = 0.000) and Ki-67 index (*p* = 0.016). As previously reported, TILs and molecular basal-like subtypes predict a high response to chemotherapy [14,21,22,23,24,25]. This can explain why immune-strong cases have a better prognosis. Immune-strong cases tend to have low proliferate activity, which might be another reason why they had a better prognosis. We also found that high immune gene expression was significantly correlated with positivity in tumor cells’ PD-L1 expression (*p* = 0.022) but not in TILs’ PD-L1 expression. This may indicate that high immune gene expression is due to immune evasion by tumor cells. TILs and PD-L1 are predictive markers of neoadjuvant chemotherapy and immunotherapy responses [6,26,27,28,29,30,31]. The close correlation between TILs’ immune gene expression and tumor cells’ PD-L1 expression indicated that the immune gene might be another predictor for chemotherapy or immune treatment.

Immune gene expression varies according to the molecular subtype, except for a luminal B case that was immune-strong. Among the remaining three subtypes, luminal A had the highest proportion (12/13, 92.3%) of immune-strong cases. The difference between the proportions of the HER2-enriched (54%) and basal-like (48.8%) groups was insignificant, whereas that between luminal A and basal-like groups was significant. As shown earlier, the prognosis of luminal A and HHER2-enriched subtypes of TNBC after chemotherapy was worse than that of the basal-like subtype. Our findings had clinical meanings. The data showed that not all triple-negative breast cancers benefit from chemotherapy. Further classification will enable some triple-negative breast cancer, such as luminal breast cancer, HER2-enriched subtype, and i-strong breast cancer to reduce the dose of chemotherapy. Just as not all recurrent TNBC and HER2-rich breast cancer must undergo mastectomy [32], for luminal type cases, HER2 or PIK3CA mutations may be present. For HEHER2-enriched patients, there might be the presence of HHER2 mutations or be HER2 low expression which could be beneficial for corresponding target medicine. For patients with i-strong, reducing chemotherapy and adding immunotherapy may achieve better survival. Combining the subtype and immune score of TNBC can enable them to receive more precise treatment. There were clinical trials, such as NSABP B-59/GBG-96-GEPARDOUZE and FUTURE, studies incorporating molecular subtype and immune signatures for therapy stratification.

This study has several limitations. First, its retrospective design may introduce selection bias, and the relatively small sample size limits the generalizability of the findings. Second, the stratification of patients based on molecular subtypes and immune scores may be affected by unaccounted confounding factors and the intrinsic heterogeneity of TNBC. Third, potential inter-assay variability in RNA-seq-derived immune and subtype scores could influence the robustness of the classification. Fourthly, the treatment era (2002–2014) of our data predates modern TNBC management, including immunotherapy and the more routine use of neoadjuvant therapy. At present, PAM50 (Prosigna) is commercially available but remains relatively costly and is not universally accessible across institutions. Similarly, the immune gene panel used in our study requires next-generation sequencing infrastructure and is not yet routinely implemented outside of research settings. At our center, neither assay is performed as part of routine clinical care for TNBC; both are currently used in research or selected clinical contexts only. Broader adoption will depend on future evidence of clinical utility, cost-effectiveness, and integration into standardized testing pathways.

## 5. Conclusions

This study revealed an intrinsic subtype-dependent impact on the prognosis of TNBC patients after adjuvant chemotherapy. Patients with intrinsic molecular subtypes other than basal-like might need different therapeutic strategies, such as endocrine or target therapies, to improve their prognosis. Immunity score was a good predictor of the prognosis of later-stage TNBC and might be a predictive marker of chemotherapy or immune therapy. The combination of molecular subtypes and immune status evaluation would be more useful for predicting the prognosis and the likelihood of benefit from chemotherapy in TNBC patients.

## Figures and Tables

**Table 1:** Patient characteristics.

Characteristics		N (%)
Age (years)		
	<50	57 (51.4%)
	≥50	54 (48.6%)
Histopathology		
	Invasive carcinoma of no special type	108 (97.3%)
	Others	3 (2.3%)
	Carcinoma of mixed type (NST + ILC)	1
	Medullary carcinoma	2
Tumor size (cm)		
	≤2.0	45 (40.5%)
	>2, ≤5 >5 NA	59 (53.2%) 6 (5.4%) 1 (0.9%)
Grade		
	Grade 1	0
	Grade 2	28 (25.2%)
	Grade 3	83 (74.8%)
Ki-67		
	+1 (<14%)	7 (6.3%)
	+2(≥14%)	104 (93.7%)
P53		
	Positive Negative NA	60 (54.1%) 50 (45.0%) 1 (0.9%)
Basal-like marker		
	Positive	96 (86.5%)
	Negative	15 (13.5%)
PAM50		
	Luminal A	13 (11.7%)
	Luminal B	1 (0.9%)
	HER2-enriched	11 (9.9%)
	Basal-like	86 (77.5%)
Lymph nodes status		
	Positive	49 (44.1%)
	Negative	57 (51.4%)
	NA	5 (4.5%)
Immune score		
	Strong	61 (55.0%)
	Weak	50 (45.0%)
Recurrent status		
	Yes	47 (42.3%)
	No	64 (57.7%)
Distant metastasis		
	Yes No	37 (33.3%) 74 (66.7%)
Living status		
	Yes	82 (73.9%)
	No	29 (26.1%)

NST, non-specialized type; ILC, invasive lobular carcinoma.

**Table 2:** Molecular subtypes and ROR.

Molecular Subtype	ROR			ER		PR		HER2	
	Low	Med	High	N	P	N	P	N	P
Luminal A	5	8	0	4	9	7	6	13	0
Luminal B	0	0	1	0	1	0	0	0	0
HER2-enriched	0	6	5	9	2	10	1	8	3
Basal-like	1	21	64	68	18	80	6	86	0

ROR: risk of recurrence; N: negative; P: positive.

**Table 3:** High immune score (i-strong) and clinicopathological features.

Clinical Parameters		No. of Patients	Immune Score I-Strong	Immune Score I-Weak	*p* Value
**Age**					
	<50	57	29	28	0.446
	≥50	54	32	22	
**Tumor size**					
	≤2 cm	45	25	20	0.965
	>2, ≤5 cm >5 cm	59 6	32 3	27 3	0.965 0.812
**Grade**					
	Grade 1 or 2	27	15	12	1.000
	Grade 3	84	46	38	
**P53**					
	Positive	60	26	34	0.702
	Negative	50	24	26	
**Ki-67 index**					
	>14%	104	54	50	0.016 *
	≤14%	7	7	0	
**PAM50 proliferation score**					
	Low or moderate High	35 76	31 30	4 46	0.000 *
**IHC-based basal-like subtype**					
	Yes	96	51	45	0.409
	No	15	10	5	
**PAM50-based basal-like subtype**					
	Yes	86	42	44	0.022 *
	No	25	19	6	
**PD-L1 expression of tumor cell**					
	Positive Negative	10 101	9 52	1 49	0.022 *
**PD-L1 expression of TILs**					
	Positive Negative	45 66	29 32	16 34	0.071
**PD1 expression of TILs**					
	Positive Negative	83 26	48 12	35 14	0.368
**TILs**					
	0 and 1 2 and 3	82 28	39 21	43 7	0.015 *
**Lymph node metastasis**					
	Yes No	49 57	28 28	21 29	0.441

ROR: risk of recurrence; N: negative; P: positive; *: *p* < 0.05.

**Table 4:** Multivariate analysis of immune score affecting recurrence.

	HR	95%CI	P (Log-Rank)
I-strong	1.421	0.581–3.478	0.442
PD1 expression of TILs	3.836	1.476–9.973	0.006
TILs score higher than 2	3.118	0.631–15.415	0.163
Lymph node metastasis	0.228	0.030–1.727	0.153

**Table 5:** Multivariate analysis of high immune score affecting mortality.

	HR	95%CI	P (Log-Rank)
I-strong	2.725	0.903–8.223	0.075
PD1 expression of TILs	3.081	1.052–9.026	0.040
TILs score higher than 2	2.691	0.289–25.031	0.384
Lymph node metastasis	0.000	0.000	0.982

## Data Availability

The datasets used and/or analyzed during the current study are available from the corresponding author upon reasonable request.

## Supplementary Materials

The following supporting information can be downloaded at https://www.mdpi.com/article/10.3390/cancers17244010/s1, Scheme S1. Sensitivity analysis of immunity score cut-off values for defining immune-strong versus immune-weak status.; Scheme S2. Kaplan–Meier analyses of disease-free and overall survival with molecular subtype, ROR and immune score and post-hoc power analysis.; Scheme S3. Disease-free and overall survival of PAM50 basal-like triple-negative breast cancers according to immune status and post-hoc power analysis.; Scheme S4. Detailed Kaplan–Meier analyses of disease-free and overall survival by molecular subtype and immune status and post-hoc power analysis.; Table S1. The immune situation in all four molecular subtypes.; Table S2. Characteristics of discordance between IHC and molecular subtype.; Table S3. Characteristics of discordance in molecular subtype of IHC basal-like but molecular subtype not.; Table S4. Multivariate analysis of immune score affecting recurrence (with non-basal/i-weak subtype).; Table S5. Multivariate analysis of high immune score affecting mortality (with non-basal/i-weak subtype).
