# Supplementary material for: PAM50 Intrinsic Subtypes and Immunity Status in Prognosis of Triple-Negative Breast Cancer: A Retrospective Cohort Study
Source: Cancers (Basel). 2025 Dec 16;17(24):4010. doi: 10.3390/cancers17244010 (PMC12731632; doi:10.3390/cancers17244010)
Supplement: Supplementary file 1 [file cancers-17-04010-s001.zip › cancers-4005755-Supplementary Materials 1.pdf]

Updated Supplementary Materials 1

Scheme S1. Sensitivity analysis of immunity score cut-off values for defining immune-strong versus immune-weak status
Page 2

Scheme S2. Kaplan–Meier analyses of disease-free and overall survival with molecular subtype, ROR and immune score and post-hoc power analysis
Page 3-10

Scheme S3. Disease-free and overall survival of PAM50 basal-like triple-negative breast cancers according to immune status and post-hoc power analysis
Page 11-14

Scheme S4. Detailed Kaplan–Meier analyses of disease-free and overall survival by molecular subtype and immune status and post-hoc power analysis
Page 15-22

Scheme S1. Sensitivity analysis of immunity score cut-off values for defining immune-strong versus immune-weak status

Immune score cutoff	35	40	45	50	55	60
Hazard ratio	1.3	1.4	1.5	1.4	1.5	1.3

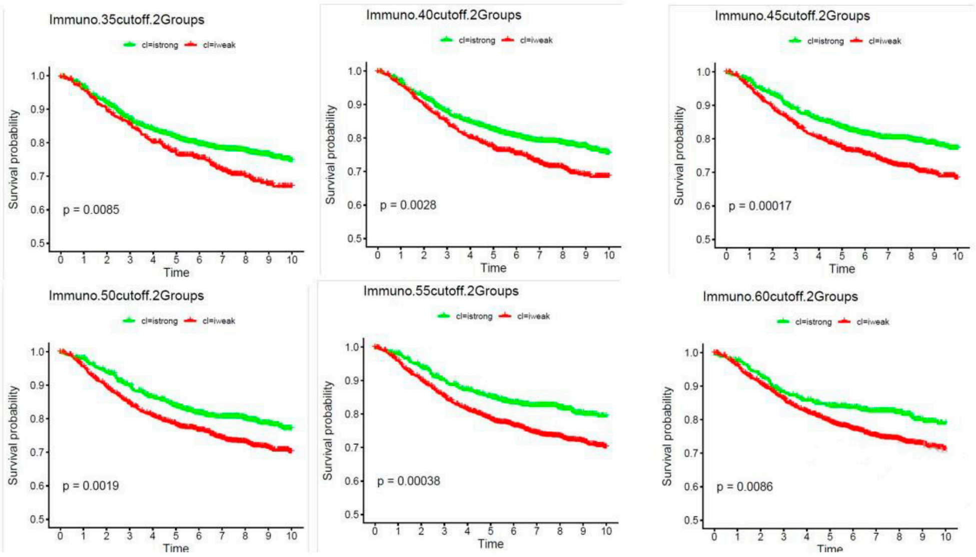

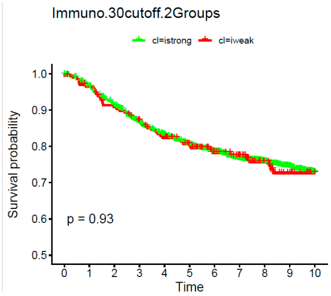

An immunity score based on the expression profile of 17 immunity genes was calculated and scaled as 0-100 according to the method published by Yang et al. Immunity scores between 35-60 were evaluated for sensitivity (supplement) that best separates immune status.

Yang B, Chou J, Tao Y, Wu D, Wu X, Li X, Li Y, Chu Y, Tang F, Shi Y *et al*: **An assessment of prognostic immunity markers in breast cancer**. *NPJ Breast Cancer* 2018, **4**:35.

Scheme S2. Kaplan–Meier analyses of disease-free and overall survival with molecular subtype, ROR and immune score and post-hoc power analysis

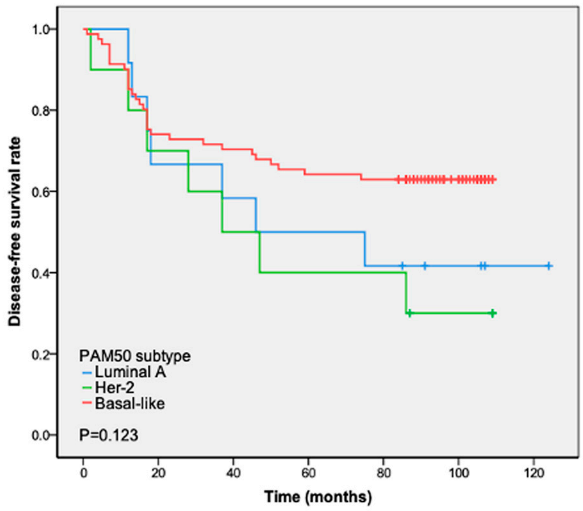

In the survival analysis, intergroup differences in DFS were not statistically significant (log-rank test, all $p > 0.05$).

Overall Comparisons

	Chi-Square	df	Sig.
Log Rank (Mantel-Cox)	4.190	2	0.123
Breslow (Generalized Wilcoxon)	2.950	2	0.229
Tarone-Ware	3.538	2	0.171

Test of equality of survival distributions for the different levels of PAM50 subtype.

Post hoc power analysis：DFS was compared among groups using the Kruskal–Wallis H test with Dunn–Bonferroni post hoc pairwise comparisons; no statistically significant differences were observed between any pair of groups (all $p > 0.05$).

Hypothesis Test Summary

	Null Hypothesis	Test	Sig.	Decision
1	The distribution of Disease-free survival time is the same across categories of PAM50 subtype.	Independent-Samples Kruskal-Wallis Test	.621	Retain the null hypothesis.

Asymptotic significances are displayed. The significance level is .050.

CI：

Means and Medians for Survival Time

	Mean[a]				Median			
			95% Confidence Interval				95% Confidence Interval	
PAM50 subtype	Estimate	Std. Error	Lower Bound	Upper Bound	Estimate	Std. Error	Lower Bound	Upper Bound
Lum A	69.833	14.043	42.310	97.357	46.000	32.909	0.000	110.502
Her-2	55.600	13.009	30.102	81.098	37.000	15.021	7.559	66.441
Basel like	76.783	4.681	67.609	85.957	.	.	.	.
Overall	81.738	4.882	72.169	91.308	.	.	.	.

a. Estimation is limited to the largest survival time if it is censored.

Median survival time and 95% CI are not available because the median survival was not reached (survival curve did not fall below 50%).

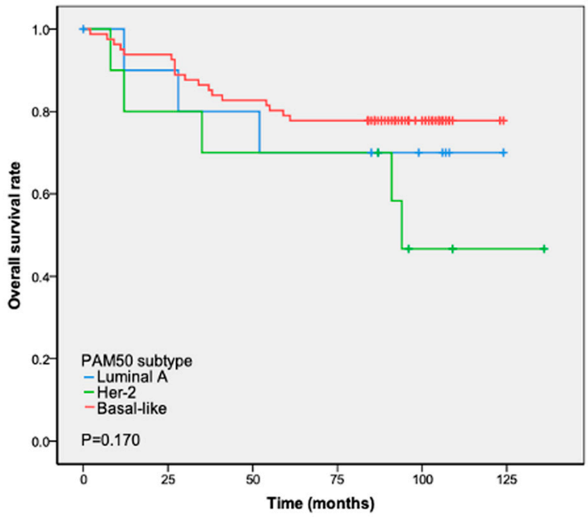

In the survival analysis, intergroup differences in OS were not statistically significant (log-rank test, all $p > 0.05$).

Overall Comparisons

	Chi-Square	df	Sig.
Log Rank (Mantel-Cox)	3.543	2	0.170
Breslow (Generalized Wilcoxon)	2.291	2	0.318
Tarone-Ware	2.809	2	0.245

Test of equality of survival distributions for the different levels of PAM50 subtype.

Post hoc power analysis：OS was compared among groups using the KWH test with Dunn–Bonferroni post hoc pairwise comparisons; no statistically significant differences were observed between any pair of groups (all $p > 0.05$).

Hypothesis Test Summary

	Null Hypothesis	Test	Sig.	Decision
1	The distribution of overall survival time is the same across categories of PAM50 subtype.	Independent-Samples Kruskal-Wallis Test	0.823	Retain the null hypothesis.

Asymptotic significances are displayed. The significance level is .050.

CI：

Means and Medians for Survival Time

PAM50 subtype	Mean[a]				Median			
			95% Confidence Interval				95% Confidence Interval	
	Estimate	Std. Error	Lower Bound	Upper Bound	Estimate	Std. Error	Lower Bound	Upper Bound
Lum A	98.545	12.797	73.462	123.628	.	.	.	.
Her-2	90.550	16.191	58.815	122.285	94.000	.	.	.
Basel like	103.549	4.355	95.013	112.085	.	.	.	.
Overall	109.924	4.536	101.033	118.814	.	.	.	.

a. Estimation is limited to the largest survival time if it is censored.

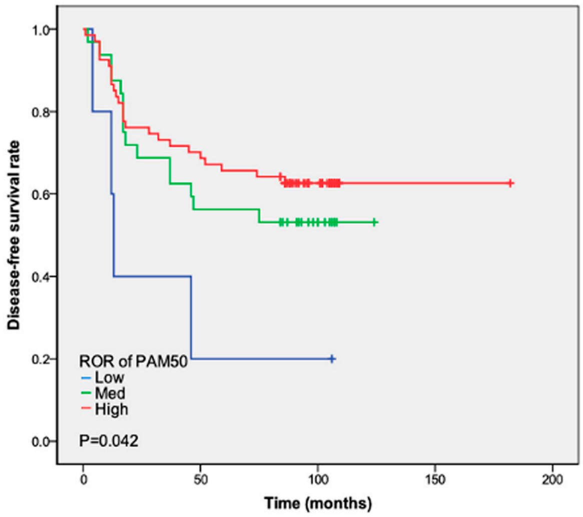

Overall Comparisons

	Chi-Square	df	Sig.
Log Rank (Mantel-Cox)	6.323	2	0.042
Breslow (Generalized Wilcoxon)	6.374	2	0.041
Tarone-Ware	6.386	2	0.041

Test of equality of survival distributions for the different levels of ROR of PAM50.

Post hoc power analysis： OS was compared among groups using the KWH test with Dunn–Bonferroni post hoc pairwise comparisons; no statistically significant differences were observed between any pair of groups (all p > 0.05).

Hypothesis Test Summary

	Null Hypothesis	Test	Sig.	Decision
1	The distribution of Disease-free survival time is the same across categories of ROR of PAM50.	Independent-Samples Kruskal-Wallis Test	0.223	Retain the null hypothesis.

Asymptotic significances are displayed. The significance level is 0.050.

CI：

Means and Medians for Survival Time

ROR of	Mean[a]	Median

PAM50			95% Confidence Interval				95% Confidence Interval	
			Lower	Upper			Lower	Upper
	Estimate	Std. Error	Bound	Bound	Estimate	Std. Error	Bound	Bound
Low	36.200	16.886	3.103	69.297	13.000	1.095	10.853	15.147
Med	77.844	8.975	60.252	95.435	.	.	.	.
High	123.691	9.376	105.315	142.068	.	.	.	.
Overall	115.570	7.716	100.446	130.693	.	.	.	.

a. Estimation is limited to the largest survival time if it is censored.

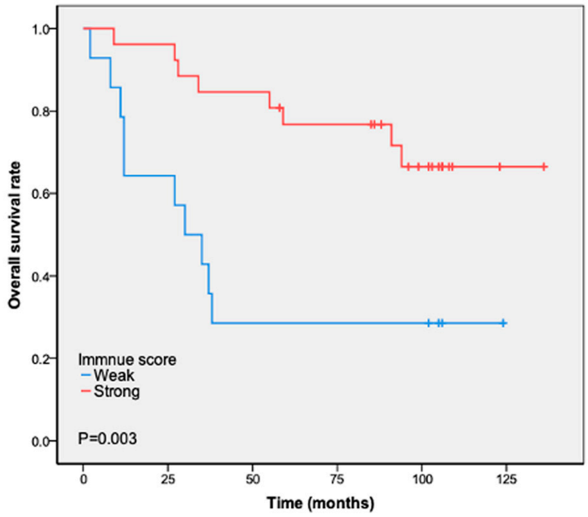

Overall Comparisons

	Chi-Square	df	Sig.
Log Rank (Mantel-Cox)	8.724	1	0.003
Breslow (Generalized Wilcoxon)	10.201	1	0.001
Tarone-Ware	9.628	1	0.002

Test of equality of survival distributions for the different levels of immune score.

Post hoc power analysis： DFS was compared among groups using the Mann-Whitney U Test (n=2) with Dunn–Bonferroni post hoc pairwise comparisons; statistically significant differences were observed between groups ($p < 0.05$).

Hypothesis Test Summary

	Null Hypothesis	Test	Sig.	Decision
1	The distribution of overall survival time is the same across categories of immune score.	Independent-Samples Mann-Whitney U Test	0.018[a]	Reject the null hypothesis.

Asymptotic significances are displayed. The significance level is 0.050.

a. Exact significance is displayed for this test.

CI:

Means and Medians for Survival Time

	Mean[a]				Median			
			95% Confidence Interval				95% Confidence Interval	
Immune score	Estimate	Std. Error	Lower Bound	Upper Bound	Estimate	Std. Error	Lower Bound	Upper Bound
Weak	50.571	12.746	25.589	75.554	30.000	7.483	15.333	44.667
Strong	108.171	8.554	91.405	124.936	.	.	.	.
Overall	89.377	8.493	72.730	106.024	.	.	.	.

a. Estimation is limited to the largest survival time if it is censored.

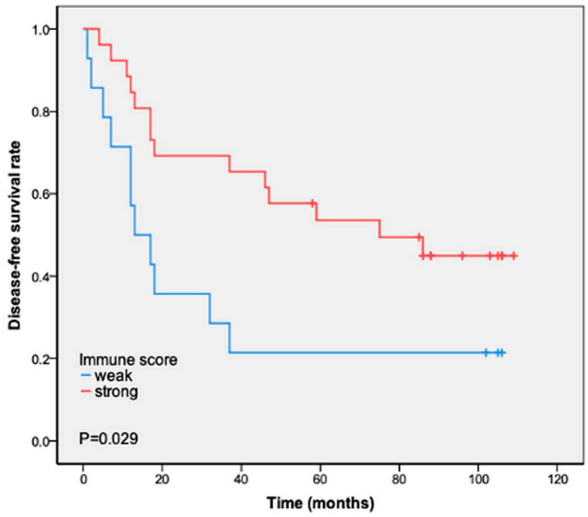

Overall Comparisons

	Chi-Square	df	Sig.
Log Rank (Mantel-Cox)	4.739	1	0.029
Breslow (Generalized Wilcoxon)	5.875	1	0.015
Tarone-Ware	5.452	1	0.020

Test of equality of survival distributions for the different levels of immune score.

Post hoc power analysis: DFS was compared among groups using the Mann-Whitney U Test with Dunn–Bonferroni post hoc pairwise comparisons; statistically significant differences were observed between groups ($p < 0.05$).

Hypothesis Test Summary

	Null Hypothesis	Test	Sig.	Decision
1	The distribution of Disease-free survival time is the same across categories of immune score.	Independent-Samples Mann-Whitney U Test	0.039[a]	Reject the null hypothesis.

Asymptotic significances are displayed. The significance level is .050.

a. Exact significance is displayed for this test.

CI:

Means and Medians for Survival Time

Immune score	Mean[a]				Median			
	Estimate	Std. Error	95% Confidence Interval		Estimate	Std. Error	95% Confidence Interval	
			Lower Bound	Upper Bound			Lower Bound	Upper Bound
Weak	33.857	10.401	13.472	54.242	13.000	4.677	3.833	22.167
Strong	67.197	8.363	50.806	83.588	75.000	30.324	15.565	134.435
Overall	55.799	7.054	41.974	69.625	37.000	11.859	13.757	60.243

a. Estimation is limited to the largest survival time if it is censored.

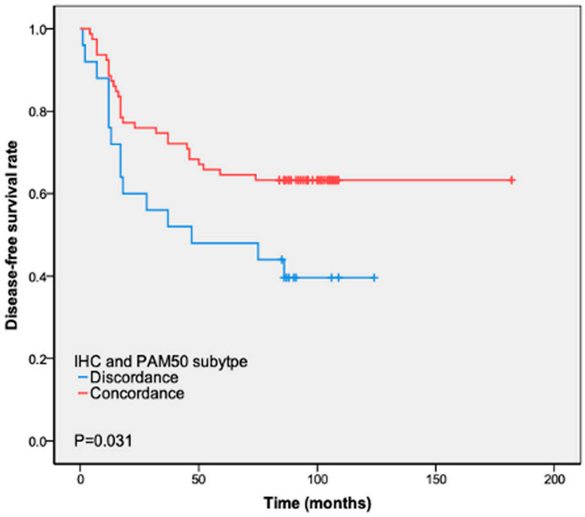

Overall Comparisons

	Chi-Square	df	Sig.
Log Rank (Mantel-Cox)	4.643	1	0.031
Breslow (Generalized Wilcoxon)	4.466	1	0.035
Tarone-Ware	4.562	1	0.033

Test of equality of survival distributions for the different levels of the concordance of IHC and PAM50 subtype.

Post hoc power analysis：DFS was compared among groups using the Mann-Whitney U Test with Dunn–Bonferroni post hoc pairwise comparisons; no statistically significant differences were observed between groups (p =0.05).

Hypothesis Test Summary

	Null Hypothesis	Test	Sig.	Decision
1	The distribution of Disease-free survival time is the same across categories of the concordance of IHC and PAM50 subtype.	Independent-Samples Mann-Whitney U Test	0.050	Retain the null hypothesis.

Asymptotic significances are displayed. The significance level is 0.050.

CI:

Means and Medians for Survival Time

the concordance of IHC and PAM50 subtype	Mean[a]				Median			
			95% Confidence Interval				95% Confidence Interval	
	Estimate	Std. Error	Lower Bound	Upper Bound	Estimate	Std. Error	Lower Bound	Upper Bound
Discordance	64.808	10.357	44.508	85.108	47.000	39.135	0.000	123.705
Concordance	124.329	8.611	107.451	141.208	.	.	.	.
Overall	115.570	7.716	100.446	130.693	.	.	.	.

a. Estimation is limited to the largest survival time if it is censored.

Scheme S3. Disease-free and overall survival of PAM50 basal-like triple-negative breast cancers according to immune status and post-hoc power analysis

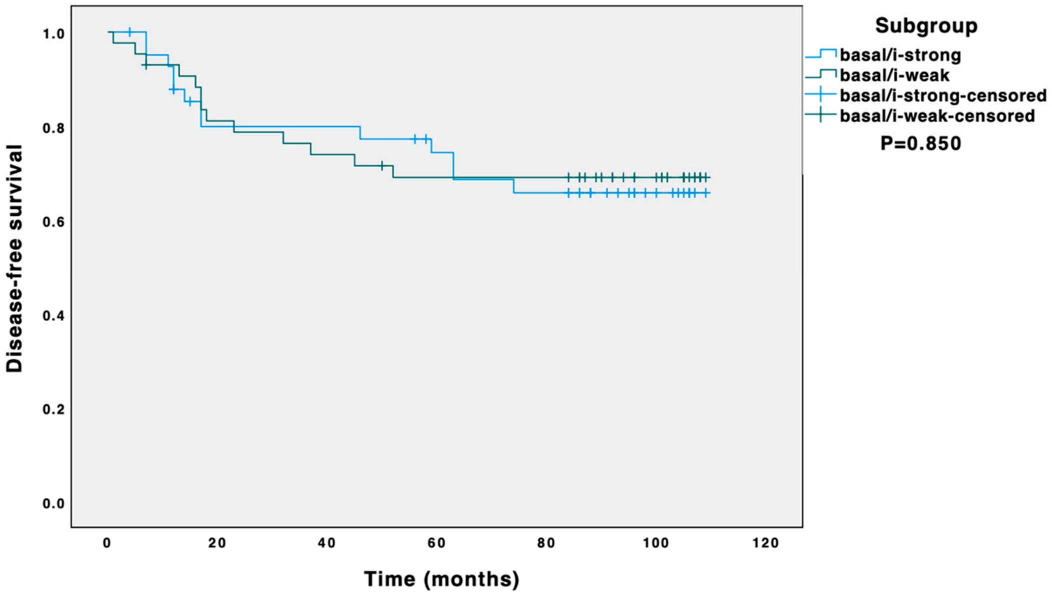

Disease-free survival of molecular basal subtype in different Immuno-groups.

Overall Comparisons

	Chi-Square	df	Sig.
Log Rank (Mantel-Cox)	.036	1	.850
Breslow (Generalized Wilcoxon)	.013	1	.909
Tarone-Ware	.022	1	.883

Test of equality of survival distributions for the different levels of Subgroup.

Post hoc analysis:

Hypothesis Test Summary

	Null Hypothesis	Test	Sig.[a,b]	Decision
1	The distribution of Disease-free survival time is the same across categories of molecular basal subtype in different Immuno-group.	Independent-Samples Mann-Whitney U Test	.165	Retain the null hypothesis.

a. The significance level is .050.

b. Asymptotic significance is displayed.

CI:

Means and Medians for Survival Time

	Mean[a]				Median			
			95% Confidence Interval				95% Confidence Interval	
Subgroup	Estimate	Std. Error	Lower Bound	Upper Bound	Estimate	Std. Error	Lower Bound	Upper Bound
basal/i-strong	82.808	6.276	70.507	95.109	.	.	.	.
basal/i-weak	82.092	6.328	69.689	94.495	.	.	.	.
Overall	82.401	4.474	73.633	91.169	.	.	.	.

a. Estimation is limited to the largest survival time if it is censored.

Case Processing Summary

			Censored	
Subgroup	Total N	N of Events	N	Percent
basal/i-strong	42	13	29	69.0%
basal/i-weak	43	13	30	69.8%
Overall	85	26	59	69.4%

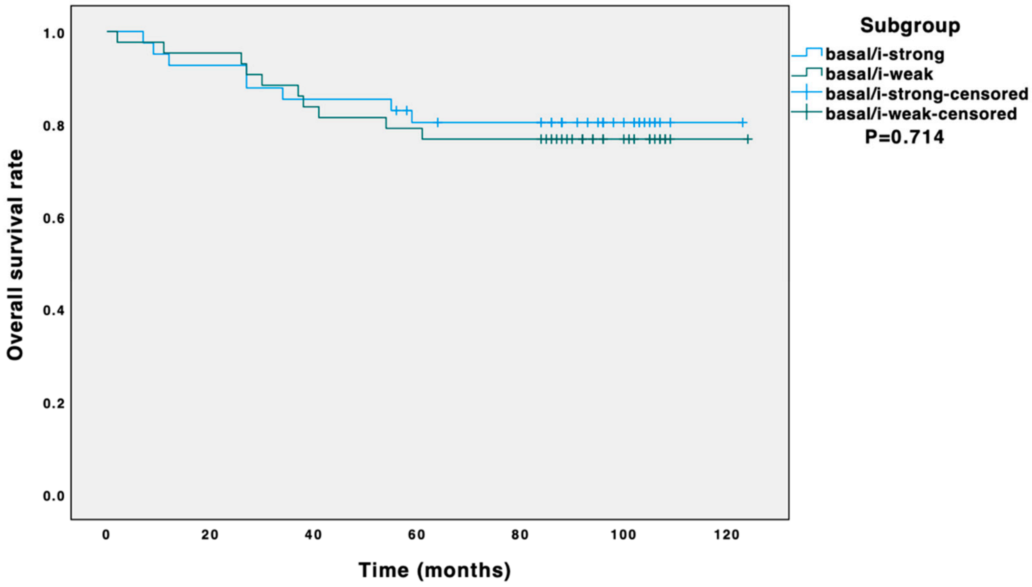

Overall survival of molecular basal subtype in different Immuno-groups.

Overall Comparisons

	Chi-Square	df	Sig.
Log Rank (Mantel-Cox)	.134	1	.714
Breslow (Generalized Wilcoxon)	.106	1	.745
Tarone-Ware	.120	1	.729

Test of equality of survival distributions for the different levels of Subgroup.

Hypothesis Test Summary

	Null Hypothesis	Test	Sig.[a,b]	Decision
1	The distribution of Overall survival time is the same across categories of molecular basal subtype in different Immuno-group.	Independent-Samples Mann-Whitney U Test	.398	Retain the null hypothesis.

a. The significance level is .050.

b. Asymptotic significance is displayed.

Means and Medians for Survival Time

	Mean[a]				Median			
			95% Confidence Interval				95% Confidence Interval	
Subgroup	Estimate	Std. Error	Lower Bound	Upper Bound	Estimate	Std. Error	Lower Bound	Upper Bound
basal/i-strong	104.512	5.992	92.768	116.256	.	.	.	.

basal/i-weak	102.767	6.012	90.983	114.552	.	.	.	.
Overall	104.013	4.268	95.648	112.379	.	.	.	.

a. Estimation is limited to the largest survival time if it is censored.

Case Processing Summary

Subgroup	Total N	N of Events	Censored	
			N	Percent
basal/i-strong	41	8	33	80.5%
basal/i-weak	43	10	33	76.7%
Overall	84	18	66	78.6%

Scheme S4. Detailed Kaplan–Meier analyses of disease-free and overall survival by molecular subtype and immune status and post-hoc power analysis

A

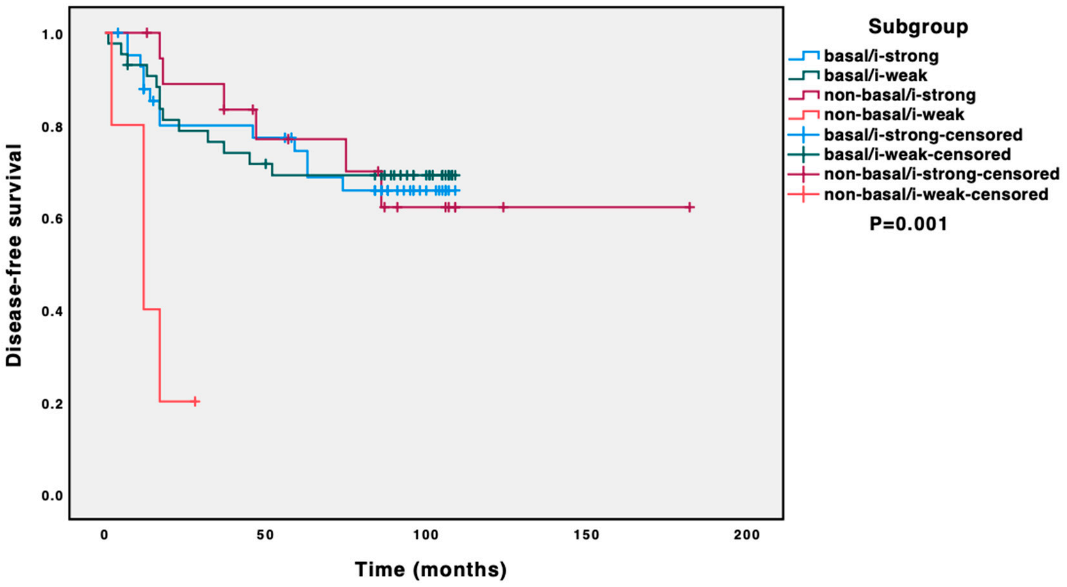

A. Disease-free survival of molecular basal or non-basal subtype in different Immuno-groups.

Overall Comparisons

	Chi-Square	df	Sig.
Log Rank (Mantel-Cox)	17.189	3	.001
Breslow (Generalized Wilcoxon)	17.529	3	.001
Tarone-Ware	17.366	3	.001

Test of equality of survival distributions for the different levels of Subgroup.

Post hoc analysis:

Hypothesis Test Summary

Null Hypothesis	Test	Sig.[a,b]	Decision

1	The distribution of Disease-free survival time is the same across categories of molecular basal or non-basal subtype in different Immuno-group.	Independent-Samples Kruskal-Wallis Test	.816	Retain the null hypothesis.

a. The significance level is .050.
b. Asymptotic significance is displayed.

CI:

Means and Medians for disease-free survival time

Subgroup	Mean[a]				Median			
	Estimate	Std. Error	95% Confidence Interval Lower Bound	95% Confidence Interval Upper Bound	Estimate	Std. Error	95% Confidence Interval Lower Bound	95% Confidence Interval Upper Bound
basal/i-strong	82.808	6.276	70.507	95.109	.	.	.	.
basal/i-weak	82.092	6.328	69.689	94.495	.	.	.	.
non-basal/i-strong	132.071	16.490	99.750	164.392	.	.	.	.
non-basal/i-weak	14.200	3.778	6.795	21.605	12.000	5.477	1.265	22.735
Overall	127.846	7.370	113.400	142.291	.	.	.	.

a. Estimation is limited to the largest survival time if it is censored.

Case Processing Summary

Subgroup	Total N	N of Events	Censored N	Censored Percent
basal/i-strong	42	13	29	69.0%
basal/i-weak	43	13	30	69.8%
non-basal/i-strong	19	6	13	68.4%
non-basal/i-weak	5	4	1	20.0%
Overall	109	36	73	67.0%

B

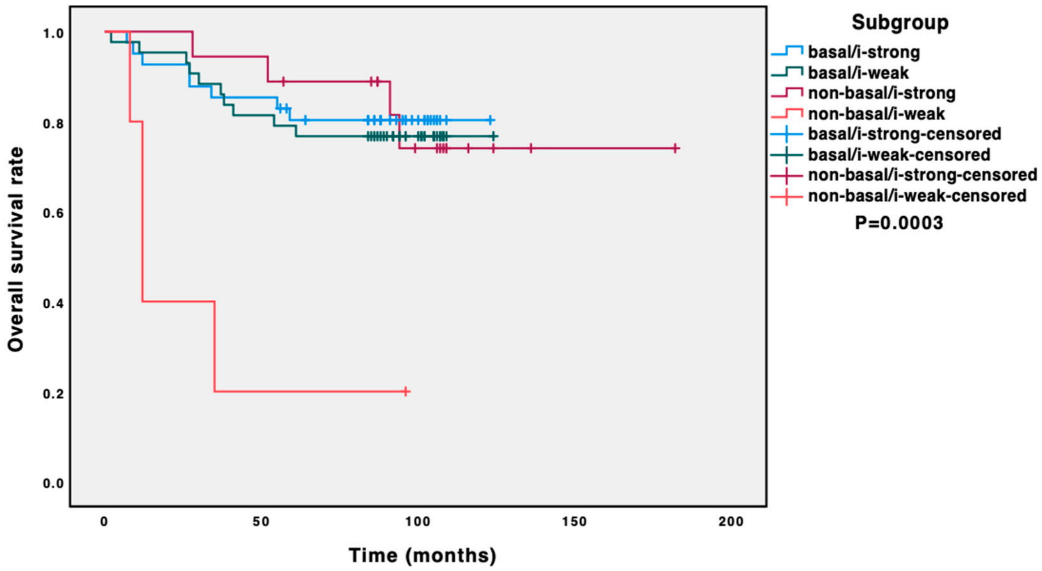

B. Overall survival of molecular basal or non-basal in different Immuno-groups.

Overall Comparisons

	Chi-Square	df	Sig.
Log Rank (Mantel-Cox)	18.751	3	.000
Breslow (Generalized Wilcoxon)	20.383	3	.000
Tarone-Ware	19.686	3	.000

Test of equality of survival distributions for the different levels of Subgroup.
Post hoc analysis:

Hypothesis Test Summary

	Null Hypothesis	Test	Sig.[a,b]	Decision
1	The distribution of Overall survival time is the same across categories of molecular basal or non-basal in different Immuno-group.	Independent-Samples Kruskal-Wallis Test	.506	Retain the null hypothesis.

Means and Medians for Survival Time

Subgroup	Mean[a]				Median			
			95% Confidence Interval				95% Confidence Interval	
	Estimate	Std. Error	Lower Bound	Upper Bound	Estimate	Std. Error	Lower Bound	Upper Bound
basal/i-strong	104.512	5.992	92.768	116.256	.	.	.	.
basal/i-weak	102.767	6.012	90.983	114.552	.	.	.	.
non-basal/i-strong	152.963	12.759	127.956	177.970	.	.	.	.
non-basal/i-weak	32.600	14.804	3.584	61.616	12.000	2.191	7.706	16.294
Overall	145.213	6.327	132.811	157.615	.	.	.	.

a. The significance level is .050.

b. Asymptotic significance is displayed.

CI:

a. Estimation is limited to the largest survival time if it is censored.

Case Processing Summary

Subgroup	Total N	N of Events	Censored	
			N	Percent
basal/i-strong	42	13	29	69.0%
basal/i-weak	43	13	30	69.8%
non-basal/i-strong	19	6	13	68.4%
non-basal/i-weak	5	4	1	20.0%
Overall	109	36	73	67.0%

C

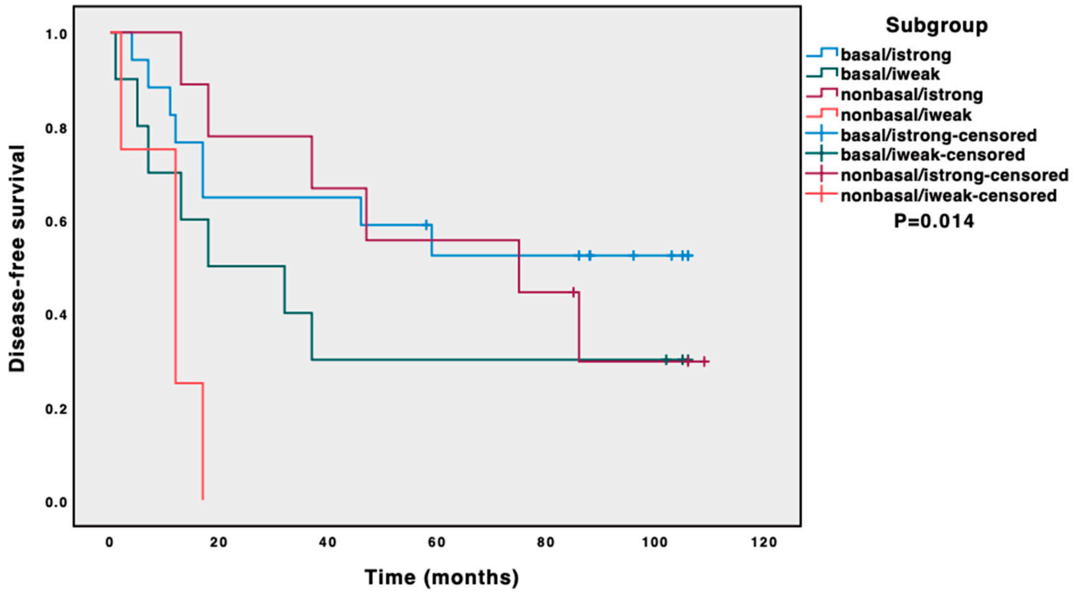

C. Disease-free survival of basal or non-basal in different Immuno-groups in IIB and advanced samples

Overall Comparisons

	Chi-Square	df	Sig.
Log Rank (Mantel-Cox)	10.677	3	.014
Breslow (Generalized Wilcoxon)	9.983	3	.019
Tarone-Ware	10.301	3	.016

Test of equality of survival distributions for the different levels of category.

Post hoc analysis:

Hypothesis Test Summary

	Null Hypothesis	Test	Sig.[a,b]	Decision
1	The distribution of disease-free survival is the same across categories of different Immuno-group in IIB and advanced samples.	Independent-Samples Kruskal-Wallis Test	.090	Retain the null hypothesis.

Means and Medians for Survival Time

category	Mean[a]				Median			
			95% Confidence Interval				95% Confidence Interval	
	Estimate	Std. Error	Lower Bound	Upper Bound	Estimate	Std. Error	Lower Bound	Upper Bound
basal/i-strong	65.987	10.684	45.047	86.927	.	.	.	.
basal/i-weak	43.100	13.451	16.736	69.464	18.000	15.021	.000	47.441
non-basal/i-strong	66.148	12.180	42.275	90.021	75.000	41.740	.000	156.810
non-basal/i-weak	10.750	3.146	4.584	16.916	12.000	4.330	3.513	20.487
Overall	55.799	7.054	41.974	69.625	37.000	11.859	13.757	60.243

a. The significance level is .050.

b. Asymptotic significance is displayed.

a. Estimation is limited to the largest survival time if it is censored.

Case Processing Summary

category	Total N	N of Events	Censored	
			N	Percent
basal/i-strong	17	8	9	52.9%
basal/i-weak	10	7	3	30.0%
non-basal/i-strong	9	6	3	33.3%
non-basal/i-weak	4	4	0	0.0%
Overall	40	25	15	37.5%

D

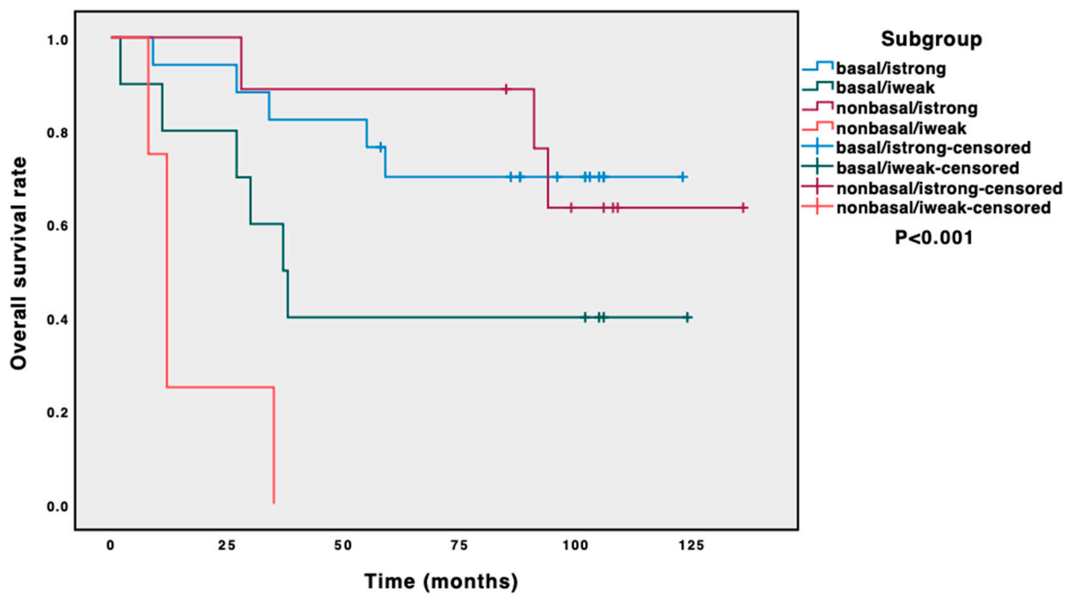

D. Overall survival of basal or non-basal in different Immuno-groups in IIB and advanced samples

Overall Comparisons

	Chi-Square	df	Sig.
Log Rank (Mantel-Cox)	19.209	3	.000
Breslow (Generalized Wilcoxon)	18.645	3	.000
Tarone-Ware	18.985	3	.000

Test of equality of survival distributions for the different levels of category.

Post hoc analysis:

Hypothesis Test Summary

	Null Hypothesis	Test	Sig.[a,b]	Decision
1	The distribution of overall survival time is the same across categories of different Immuno-group in IIB and advanced samples.	Independent-Samples Kruskal-Wallis Test	.023	Reject the null hypothesis.

a. The significance level is .050.

b. Asymptotic significance is displayed.

Pairwise Comparisons of category

Sample 1 vs Sample 2	Test Statistic	Std. Error	Std. Test Statistic	Sig.	Adj. Sig.[a]
non-basal/i-weak vs basal/i-weak	11.700	6.910	1.693	.090	.543
non-basal/i-weak vs basal/i-strong	15.632	6.491	2.408	.016	.096
non-basal/i-weak vs non-basal/i-strong	20.806	7.019	2.964	.003	.018
basal/i-weak vs basal/i-strong	3.932	4.655	.845	.398	1.000
basal/i-weak vs non-basal/i-strong	-9.106	5.367	-1.697	.090	.539
basal/i-strong vs non-basal/i-strong	-5.173	4.815	-1.074	.283	1.000

Means and Medians for Survival Time

category	Mean[a]				Median			
			95% Confidence Interval				95% Confidence Interval	
	Estimate	Std. Error	Lower Bound	Upper Bound	Estimate	Std. Error	Lower Bound	Upper Bound
basal/i-strong	97.333	9.902	77.926	116.740	.	.	.	.
basal/i-weak	64.100	15.806	33.120	95.080	37.000	6.325	24.604	49.396
non-basal/i-strong	112.952	11.996	89.441	136.464	.	.	.	.
non-basal/i-weak	16.750	6.156	4.684	28.816	12.000	1.732	8.605	15.395
Overall	89.377	8.493	72.730	106.024	.	.	.	.

Each row tests the null hypothesis that the Sample 1 and Sample 2 distributions are the same.
Asymptotic significances (2-sided tests) are displayed. The significance level is .050.
a. Significance values have been adjusted by the Bonferroni correction for multiple tests.

CI:

a. Estimation is limited to the largest survival time if it is censored.

Case Processing Summary

category	Total N	N of Events	Censored	
			N	Percent
basal/i-strong	17	5	12	70.6%
basal/i-weak	10	6	4	40.0%
non-basal/i-strong	9	3	6	66.7%
non-basal/i-weak	4	4	0	0.0%
Overall	40	18	22	55.0%
