# Supplementary material for: PAM50 Intrinsic Subtypes and Immunity Status in Prognosis of Triple-Negative Breast Cancer: A Retrospective Cohort Study
Source: Cancers (Basel). 2025 Dec 16;17(24):4010. doi: 10.3390/cancers17244010 (PMC12731632; doi:10.3390/cancers17244010)
Supplement: Supplementary file 1 [file cancers-17-04010-s001.zip › cancers-4005755-Supplementary Materials 2.pdf]

Updated Supplementary Materials 2

Table S1. The immune situation in all four molecular subtypes

Table S2. Characteristics of discordance between IHC and molecular subtype

Table S3. Characteristics of discordance in molecular subtype of IHC basal-like but molecular subtype not

Table S4. Multivariate analysis of immune score affecting recurrence (with non-basal/ i-weak subtype)

Table S5. Multivariate analysis of high immune score affecting mortality (with non-basal/ i-weak subtype)

Table S1. The immune situation in all four molecular subtypes.

	Basal-like	Luminal A	Luminal B	Her2-enriched
i-strong	43	12	1	6
i-weak	43	1	0	5
Total	86	13	1	11

Table S2. Characteristics of discordance between IHC and molecular subtype.

Basal-like subtype	IHC-based	PAM50 subtype
concordance	78	78
Discordance	18	8
Total	96	86

Table S3. Characteristics of discordance in molecular subtype of IHC basal-like but molecular subtype not.

Characteristic			N / (%)
**Molecular subtype**			
Luminal A			7 (38.9%)
Her-2			11 (61.1%)
overall			18 (100.0%)
**IHC surrogated subtype**			
Luminal A			7
	Ki-67	<30%	4
		30-50%	1
		>30%	2[a]
Her-2			11
	P53	1	5
		0	6
	Ki-67	<30%	3
		30-50%	6
		>50	2

Table S4. Multivariate analysis of immune score affecting recurrence (with non-basal/ i-weak subtype).

	HR	95%CI	P (Log-Rank)
I-strong	2.138	0.649-7.042	0.211
Non-basal/ I-weak	0.272	0.060-1.239	0.092
PD1 expression of TILs	3.180	1.066-9.488	0.038
TILs score higher than 2	2.714	0.291-25.33	0.381
Lymph node metastasis	0.000	0.000	0.984

Table S5. Multivariate analysis of high immune score affecting mortality (with non-basal/ i-weak subtype).

	HR	95%CI	P (Log-Rank)
I-strong	0.855	0.321-2.280	0.755
Non-basal/ I-weak	2.746	0.650-11.597	0.169
PD1 expression of TILs	0.267	0.102-0.696	0.007
TILs score higher than 2	0.308	0.062-1.522	0.149
Lymph node metastasis	4.229	0.557-32.107	0.163
